# Identification of apolipoprotein E-derived amyloid within cholesterol granulomas of leopard geckos (*Eublepharis macularius*)

**DOI:** 10.1038/s41598-024-64643-y

**Published:** 2024-06-14

**Authors:** Mitsuhiro Ikeda, Hirotaka Kondo, Tomoaki Murakami, Susumu Iwaide, Yoshiyuki Itoh, Hisashi Shibuya

**Affiliations:** 1 Laboratory of Veterinary Pathology, Department of Veterinary Medicine, College of Bioresource, Nihon University, Kanagawa, Japan; 2 Laboratory of Veterinary Toxicology, Tokyo University of Agriculture and Technology, Tokyo, Japan; 3 Smart-Core-Facility Promotion Organization, Tokyo University of Agriculture and Technology, Tokyo, Japan

**Keywords:** Inflammation, Proteomics

## Abstract

Apolipoprotein E (ApoE) is involved in cholesterol transport among cells and also plays an important role in amyloid formation, co-depositing with amyloid fibrils in various types of amyloidosis. Although the in vivo amyloidogenicity of ApoE has not been previously demonstrated, this study provides evidence of ApoE amyloidogenicity in leopard geckos (*Eublepharis macularius*), belonging to the class Reptilia. Histologically, amyloid deposits were localized within cholesterol granulomas and exhibited positive Congo red staining, with yellow to green birefringence under polarized light. On mass spectrometry-based proteomic analysis, ApoE was detected as a dominant component of amyloid; of the full length of the 274 amino acid residues, peptides derived from Leu185-Arg230 were frequently detected with non-tryptic truncations. Immunohistochemistry with anti-leopard gecko ApoE antibody showed positive reactions of amyloid deposits. These results show that ApoE is an amyloid precursor protein within the cholesterol granulomas of leopard geckos. Although further investigations are needed, the C-terminal region of ApoE involved in amyloid formation is a lipid-binding region, and there should be a relationship between amyloidogenesis and the development of cholesterol granulomas in leopard geckos. This study provides novel insights into the pathogenesis of ApoE-related diseases.

## Introduction

Amyloidosis is a protein misfolding disease characterized by amyloid fibril deposition in tissues and classified by amyloid precursor proteins and deposited sites^1,2^. To date, 42 types of amyloidosis have been identified in humans and nearly 20 types in animals^1,3–9^. Notably, some amyloid precursor proteins in animals, such as α-S2 casein and lipopolysaccharide-binding protein, have not been proven to have in vivo amyloidogenicity in humans^7,10^. Since such amyloids may also deposit in humans, it is important to identify novel amyloid proteins in animals and elucidate the mechanism of deposition. Though there are numerous reports of amyloidoses in mammals and birds, there are only a few reports of them in reptiles (Table 1)^11–15^. Furthermore, amyloid precursor proteins have never been identified in reptilian amyloidosis. A comparative pathological study using reptile species is important, considering they are closer relatives to mammals than birds^16^. Studying amyloidosis in reptiles using proteomic analysis could provide new insights into the mechanisms of amyloidogenesis.

**Table 1:** Literature review of amyloidosis in reptiles.

Species affected	Summary of case	Reference
African tiger snake (*Telescopus semiannulatus*)	Amyloid deposition sites: spleen, testicular interstitium, and blood vessel walls of various tissues Concurrent lesions: disseminated mycobacteriosis and a hepatic biliary cystadenocarcinoma Immunohistochemistry: negative for amyloid A	^11^
Hermann’s tortoises (*Testudo hermanni*)	30 of a group of 52 tortoises Amyloid deposition sites: vascular walls and splenic parenchyma Isolated Salmonella in 14 of 30 amyloidosis cases	^12^
Central American boa (*Constrictor imperator*) Brown tree boa *(Boa enydris enydris*)	Amyloid deposition sites: Bowman’s capsule of the kidney and spleen, respectively No information about birefringence was described in the Congo red-positive area	^13^
Spiny-tailed iguana (*Ctenosaura acanthura*) American alligator (*Alligator mississippiensis*)	No information about diagnostic criteria was documented Homogeneous materials were observed in glomeruli and spleen, respectively	^14^

Apolipoprotein E (ApoE), a member of the lipid-binding protein (apolipoprotein) family, mediates cholesterol metabolism and has been well-studied as a protein closely involved in the pathogenesis of Alzheimer’s disease^17–20^. ApoE is known to co-deposit with various types of amyloid, including amyloid-β in Alzheimer’s disease, and it is referred to as an amyloid signature protein together with other proteins having similar properties, such as heparan sulfate proteoglycans and serum amyloid P component^1,19–23^. Amyloid signature proteins are native proteins that are not amyloid proteins but co-localize with various amyloid fibrils. Among amyloid signature proteins, apolipoprotein A-I (ApoAI) and apolipoprotein A-IV (ApoAIV) have been found to exhibit amyloidogenic properties in humans and animals^8,9,24,25^. The amyloidogenic property of ApoE has also been reported in vitro^26^, but it has not yet been demonstrated in vivo.

As reptiles are becoming popular as exotic pets, there is a growing demand for veterinary medicine worldwide. The leopard gecko (*Eublepharis macularius*) is an insectivorous and nocturnal gecko that inhabits rocky deserts in Afghanistan, India, and Pakistan^27^. It is one of the most popular species of captive reptiles because of its ease of handling and various attractive color morphs. This study found amyloid deposits within cholesterol granulomas in leopard geckos and identified apolipoprotein E (ApoE) as a novel amyloidogenic protein by proteomic analysis. By establishing the novel role of ApoE in amyloidosis, this study aimed to bring a fresh perspective to the comparative pathology of ApoE function and contributes to the future elucidation of the pathogenesis of cholesterol granulomas in leopard geckos.

## Results

### Pathological features of amyloid deposits within cholesterol granulomas

**Figure 1:**
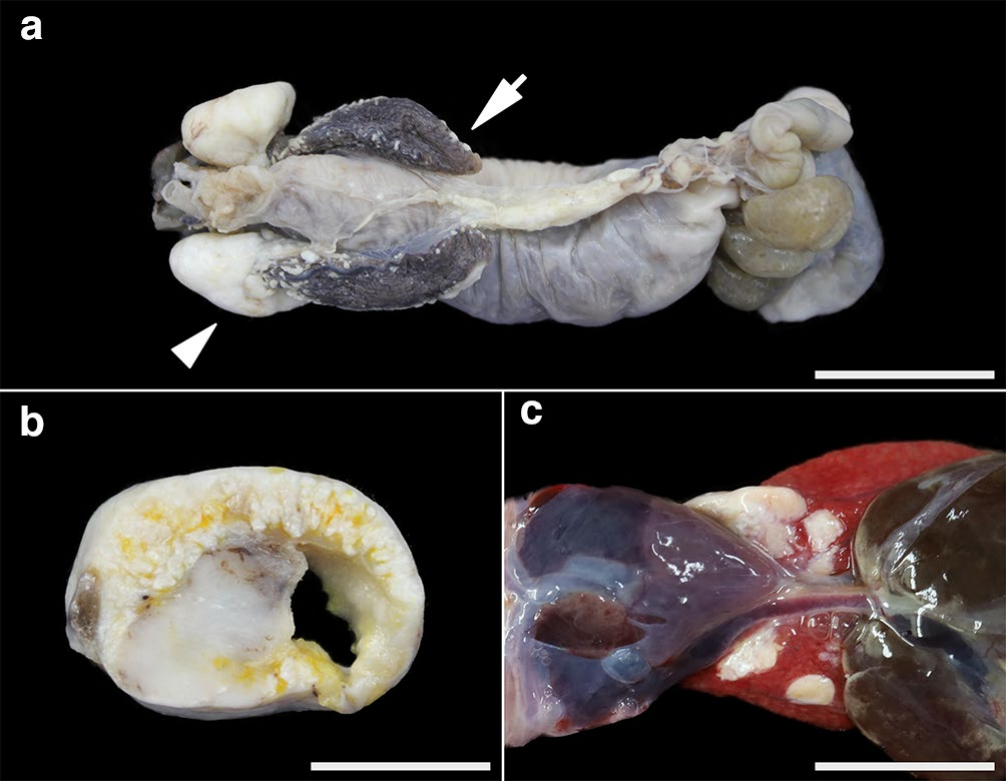
(**a**–**c**) Gross findings of cholesterol granulomas in leopard geckos. (**a**) There are multifocal white masses on the cranial aspect of the lungs (arrowhead), and smaller nodules are distributed on the caudal regions (arrows). Animal 7, formalin-fixed tissue, bar = 1.75 cm. (**b**) The cross-section of the white mass is mottled white to yellow and multinodular with an irregular central cavity. Animal 7, formalin-fixed tissue, bar = 1.2 cm. (**c**) Several tan nodules are present on the serosa of the lungs. Animal 8, bar = 0.7 cm.

**Figure 2:**
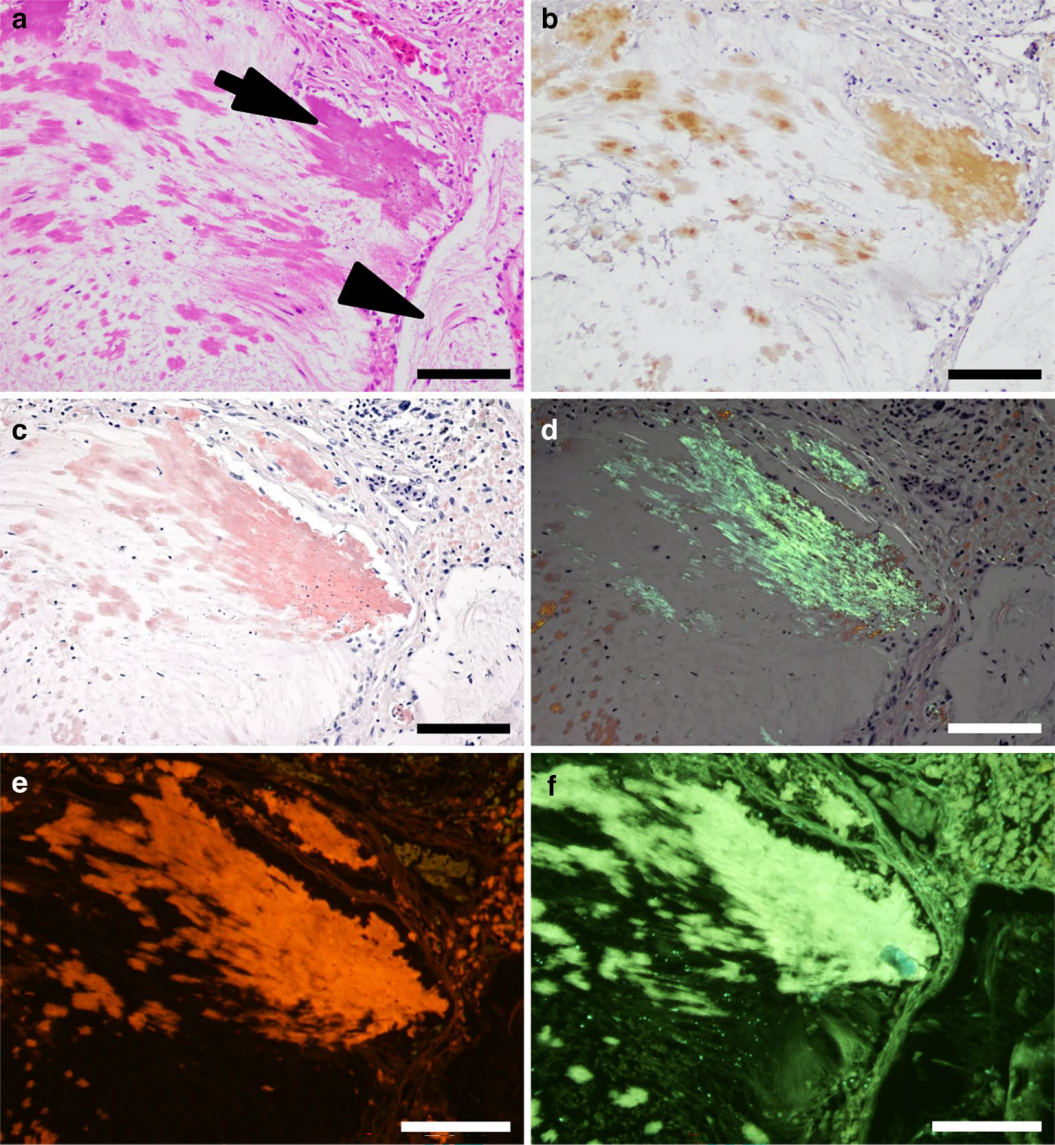
(**a**–**f**) Histopathological and immunohistochemical findings of amyloid deposits within cholesterol granulomas of a leopard gecko (Animal 5). Bars = 100 μm. (**a**) Homogeneous eosinophilic materials (arrows) are observed around the accumulation of cholesterol crystals (arrowhead) on hematoxylin and eosin staining. (**b**) Amyloid deposits are immunolabeled with anti-leopard gecko apolipoprotein E antibodies. (**c**) Amyloid deposits show positive staining for Congo red. (**d**) Congophilic areas exhibit yellow to green birefringence under polarized light. (**e**) The deposits on Congo red-stained specimen are strongly illuminated under fluorescence microscopy. (**f**) The deposits are positive for thioflavin S and exhibited strong pale-green fluorescence under blue-violet excitation.

Nine animals, seven females (78%) and two males (22%), were used in the present study. On gross examination, variably-sized, multifocal white masses were seen on the serosal surface of the lungs in animals 7 and 8 (Fig. 1). In animals 1–6 and 9, though whole gross findings were not available, they had white masses in formalin-fixed paraffin-embedded (FFPE) blocks. The masses were located in the lungs in all animals and occasionally adhered to the coelomic membrane or other organs. Histologically, the above masses were composed of an accumulation of cholesterol clefts mixed with foamy macrophages and multinucleated giant cells, which were consistent with cholesterol granulomas (Fig. 2, Supplementary Figs. S1–S4). In animal 8, only tiny cholesterol granulomas were observed in the cranial region of the lungs, and they were not present in other pulmonary areas. In the lesions, multifocal eosinophilic, amorphous, and homogeneous materials were seen in one of five biopsy samples (animal 4; Supplementary Fig. S1) and all necropsy samples (animals 5–8; Fig. 2, Supplementary Figs. S2–S4). The materials were positive for Congo red, with yellow to green birefringence under polarized light, identified as amyloid (Fig. 2, Supplementary Figs. S1–S4). Amyloid deposits were observed in the vascular walls and the fibrous stroma within cholesterol granulomas. In addition, amyloid deposits were observed in the smooth muscle of the pulmonary septa in animal 8 (Supplementary Fig. S4). In animal 5, mineralization was observed around the amyloid deposits. No amyloid deposits were detected in areas other than cholesterol granulomas in all animals with amyloid deposits. Other than cholesterol granulomas, there were no specific concurrent lesions associated with amyloid deposition. Histopathological examination of the brain was performed only in animal 8, but lesions were not seen in the brain.

With fluorescence microscopy, Congo red-stained materials were illuminated in bright red by green excitation (Fig. 2, Supplementary Figs. S1–S4). The deposits were also positive for thioflavin S and exhibited strong pale-green fluorescence under blue-violet excitation (Fig. 2, Supplementary Figs. S1–S4).

### Mass spectrometry-based proteomic analysis

**Figure 3:**
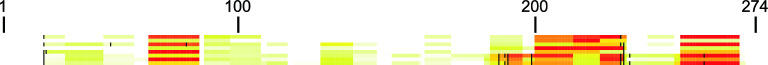
Mapping of the detected peptides in apolipoprotein E. The laser-microdissected amyloid samples from animals 4–8 are presented in the upper rows, and the three needle-microdissected amyloid samples from animal 7 are presented in the lower rows. When the total number of detected peptides increases, the color becomes redder. Of the full length of the 274 amino acid residues, Leu185–Arg230 is frequently detected. Black vertical lines mark the positions of non-tryptic truncation sites.

Amyloid deposits were microdissected from FFPE sections using laser microdissection (LMD) or 30-gauge needles and analyzed by liquid chromatography-tandem mass spectrometry (LC–MS/MS) to identify the major protein component. MS/MS results are summarized in Table 2, and all data are shown in Supplementary Tables S1–S4 online. In all of animals 4–8, ApoE was detected at the prominent values for both the number of detected peptides and the exponentially modified protein-abundance index (emPAI: the relative quantitative value of the Mascot algorithm). Serum amyloid P-component, histone, and vitronectin, which have been reported as amyloid signature proteins in humans and some animal species^1,21,22,28^, were detected at low levels in some samples, proving the presence of amyloid fibrils in the microdissected samples. Keratin 5, previously reported as an amyloid precursor protein in dogs^4^, was detected in animals 4 and 5, but at much lower levels than ApoE. The number of ApoE-derived peptides detected in animals 4–8 by mass spectrometry was mapped schematically to identify the amyloidogenic regions of ApoE (Fig. 3). Of the full length of the 274 amino acid residues, Glu63–Arg83, Leu185–Arg230, and Glu249–Lys268 were frequently detected (Fig. 3). Non-tryptic truncations often occurred at the C-terminal side of His228 and Phe229 in most animals. In the samples microdissected using 30-gauge needles, non-tryptic digestion was more frequently observed at the C-terminal side of Pro187, Leu189, Ser190, Gln198, His228, Phe229, and Gly231 due to the collection of a larger amount of samples. No peptides were detected at Met1–Ala18 in all samples.

**Table 2:** The list of proteins from laser-microdissected amyloid samples on proteomic analysis.

Accession	Protein description	Animal number									
		4		5		6		7		8	
		Peptides	emPAI	Peptides	emPAI	Peptides	emPAI	Peptides	emPAI	Peptides	emPAI
XP_054855853.1	Apolipoprotein E	41	4.48	21	1.23	85	17.23	60	4.48	62	8.05
XP_054849677.1	Serum amyloid P-component	–	–	–	–	–	–	–	–	7	0.45
XP_054848254.1	Collagen alpha-1(I) chain	2	0.05	–	–	5	0.07	–	–	1	0.02
XP_054859234.1	Keratin, type II cytoskeletal 5	2	0.06	2	0.06	–	–	–	–	–	–
XP_054826898.1	Protein Daple isoform X1	5	0.01	5	0.01	4	0.01	4	0.01	5	0.01
XP_054848662.1	Histone H4	–	–	–	–	–	–	–	–	1	0.3
XP_054858047.1	Vitronectin	–	–	–	–	–	–	3	0.13	–	–
XP_054844790.1	Histone H2B 7	–	–	–	–	1	0.22	–	–	1	0.22

Proteins were detected in at least two cases of the five animals, and amyloid signature and precursor proteins are listed. Trypsin, used for digestion, was excluded. The number of peptides indicates the count of peptides derived from each protein detected by proteomic analysis.

*emPAI* exponentially modified protein abundance index, – not detected.

### Immunohistochemical findings of amyloid deposits

A new antibody targeting Ser190–Gln205, the predicted amyloidogenic region of leopard gecko ApoE (described later), was developed and used for immunohistochemistry. Amyloid deposits in animals 4–8 had positive reactivity to anti-leopard gecko ApoE antibody (Fig. 2, Supplementary Figs. S1–S4). These amyloid deposits were negative for cytokeratin 5.

## Discussion

In this study, amyloid deposition was found in cholesterol granulomas of leopard geckos. Mass spectrometry-based proteomic analysis detected ApoE as a major component of amyloid deposits, and immunohistochemistry also confirmed this result. These results indicate that ApoE-derived amyloid was deposited within cholesterol granulomas of leopard geckos. ApoE has been known to be co-deposited with amyloid fibrils as an amyloid signature protein in human and animal amyloidosis^1,3–7,21,22^. To date, whereas the amyloidogenicity of ApoE has not been demonstrated in vivo, various apolipoproteins such as serum amyloid A, ApoAI, apolipoprotein A-II, ApoAIV, apolipoprotein C-II, and apolipoprotein C-III (ApoCIII) have been identified as amyloid precursor proteins in humans and/or animals^2,5,8,9,29–32^. Since ApoAI and ApoAIV also have properties as amyloid signature proteins^1,21,22^, we need to be careful to diagnose ApoAI and ApoAIV amyloidosis. To diagnose ApoAIV amyloidosis, it is necessary that ApoAIV be detected at high levels, and that other amyloid precursor proteins be absent on proteomic analysis^9,32^. This study detected ApoE at very high scores in all animals, and other amyloidogenic proteins, including keratin 5 detected at low levels, were excluded^4^. Therefore, we concluded that ApoE is not an amyloid signature protein but an amyloid precursor protein within cholesterol granulomas of leopard geckos.

In this study, peptides derived from the C-terminus of ApoE were predominantly detected within amyloid deposits, and these peptides were often truncated independent of tryptic digestion. In various amyloidoses, fragmentation of the precursor protein is known to trigger amyloid formation^23^. The detection of partial peptides derived from non-tryptic digestion in ApoE may indicate that these peptides constitute amyloid fibrils in vivo. The C-terminal fragment of ApoE has been found to co-purify with amyloid β from human senile plaques^26^. In addition, this C-terminal fragment from recombinant ApoE forms amyloid fibrils in vitro^26^. The present study demonstrated that ApoE has the amyloidogenic property not only in vitro, but also in vivo. The C-terminal domain of ApoE features a large exposed hydrophobic surface that is responsible for triggering interactions with various binding partners, such as lipids and amyloid β peptides^19,33^. In ApoE, the N-terminal domain consisting of four-helix bundles demonstrates higher stability, whereas the C-terminal domain exhibits low stability^34^. Intriguingly, full-length ApoE has been negative for Congo-red, whereas the C-terminus of ApoE exhibited yellow to green birefringence in the previous report^26^. Intensive non-tryptic cleavage was observed at His228 and Phe229, indicating the involvement of Leu185–Arg230 as an amyloidogenic region in leopard gecko ApoE. Therefore, we suggest that leopard gecko ApoE forms amyloid as a result of alterations in the conformational stability of the C-terminal domain via truncation.

There is only one previous report of cholesterol granulomas in a leopard gecko, in which the presence of amyloid was not mentioned^35^. The occurrence of cholesterol granulomas is higher in females than in males in reptiles^36,37^. The same situation was seen in the present study, but the reason for the sex predilection remains unclear. In the present study, the incidence of amyloid depositions within cholesterol granulomas was 20% on biopsy (1/5) and 100% on necropsy (4/4) cases. The reason for the higher ratio of amyloid deposition on necropsy than on biopsy is considered to be the larger sample amount in necropsy cases. In the previous report, the animal had a mass occupying the coelomic cavity and several tiny masses on the lung surfaces, pericardium, and liver^35^. In the present study, cholesterol granulomas were consistently located in the lungs of all animals, with observations restricted to the lungs as tiny nodules in animal 8. These findings indicate that the onset of cholesterol granulomas with ApoE amyloidosis in leopard geckos occurs initially in the lungs.

ApoE is involved in cholesterol transport among cells and has an important role in lipid metabolism^17,18^. Indeed, ApoE-deficient transgenic mice do not have normal cholesterol metabolism and develop cholesterol granulomas^38–40^. ApoE is produced not only in the hepatocytes, but also in the lung cells^41^. In the lung, ApoE is secreted by alveolar macrophages, type I and type II alveolar epithelial cells, and pulmonary artery smooth muscle cells^41^. In the present study, the positive immunoreactions for ApoE were consistent only with amyloid deposits, whereas the normal pneumocytes were negative for ApoE, indicating low to normal expression of ApoE in pneumocytes. Among apolipoproteins, ApoE and ApoAI were found to be involved in lung diseases^42^. There may be a relationship between the misfolding of ApoE and the failure of lipid metabolism of the lungs in leopard geckos; however, the linkage remains unclear, and further analysis is required. We hypothesize that, for some reason, the binding affinity between ApoE and lipids decreases, leading to the conformational instability of ApoE and subsequent misfolding. Other apolipoproteins, such as ApoAI and ApoCIII, exhibit lower conformational stability and acquire amyloidogenic properties in lipid-free states^5,30,43^. The crucial site for lipid binding in human ApoE is the C-terminal one-third, where amphipathic α-helices are formed^20,23,44^. The amphipathic α-helix, a key motif for binding lipid surfaces in apolipoproteins, has been suggested to possess a high inherent propensity for self-association and amyloid formation^20,23,44^. ApoE and ApoAI belong to the same gene family and have similar tertiary structures, although the N-terminal helix bundle in ApoAI is less organized and unstable than that in ApoE^45^. In other members of the exchangeable apolipoproteins, such as ApoAIV, the mechanism for lipid binding is a thought to have a similar feature where the N-terminus opens after the C-terminus binds to lipids, as ApoE and ApoAI behave similarly^45^. Considering that Leu185–Arg230 could be an amyloidogenic area, it is hypothesized that the reduced binding activity of ApoE to lipids on its C-terminal domain results in the development of cholesterol granulomas and amyloidosis. In view of the assumed relationship between ApoE-derived amyloid and cholesterol granulomas in leopard geckos, further analysis will be needed to demonstrate the pathogenesis.

In conclusion, this is the first study to show the in vivo amyloidogenicity of ApoE, a protein implicated in various amyloidotic pathologies, from the unique perspective of reptilian pathology. By elucidating the amyloidogenic properties of ApoE, the present study provides a new perspective on the role of ApoE in the pathogenesis of amyloidosis, as well as contributing to our understanding the pathogenesis of cholesterol granuloma in leopard geckos.

## Methods

All animals were considered clinical samples collected by veterinarians or veterinary technicians for diagnostic purposes, and materials did not require local ethics committee approval. The anti-ApoE antibody was purchased from Cosmo Bio Co., Ltd. and produced in accordance with the protocols of the animal experiment committee of Cosmo Bio Co., Ltd. The procedure was conducted using the following reference numbers: Catalog number AF23052261-001, Order number PEP13287. We confirm that all methods were carried out following relevant guidelines, regulations, and the ARRIVE guidelines in the laboratory (AL18BRS045-3) approved by Nihon University.

### Materials

Nine leopard geckos with cholesterol granulomas, including five biopsy cases and four necropsy cases, were analyzed. Detailed information on each case is summarized in Supplementary Table S5 online. Autopsies were performed at several animal hospitals for cases 5–7 and at Nihon University for case 8. In case 6, the histopathological examination was limited because only selected tissue samples were submitted. The samples were fixed in 10% neutral-buffered formalin. After trimming, the tissue samples were routinely processed and embedded in paraffin.

### Histopathological examination

For histopathological examination, FFPE blocks were sectioned at a thickness of 5 μm and stained with hematoxylin and eosin (H&E) and Congo red in all animals. In addition, sections stained with Congo red were examined under polarized microscopy.

As described previously, thioflavin S and Congo red-stained sections were examined with fluorescence microscopy (BX51, Olympus, Tokyo, Japan) equipped with a U-MWBV2 filter (λex 400–440 nm; dichroic mirror, 455 nm; λem 475 nm) for thioflavin S-stained sections and a U-MWIG filter set (λex 520–550 nm; dichroic mirror, 565 nm; λem 580 nm) for Congo red-stained specimens^46,47^. Fluorescence images were captured using a complementary metal–oxide–semiconductor (CMOS) camera (WRAYCAM-VEX120; Wraymer, Osaka, Japan).

### Mass spectrometry-based proteomic analysis

Proteomic analysis was performed on animals 4–8. FFPE specimens with amyloid deposits were cut into 5-μm-thick sections on polyethylene naphthalate membrane glass slides and stained with Congo red. Congo red-positive areas of 100,000–550,000 μm^2^ were collected by LMD System LMD7000 (Leica Microsystems, Wetzlar, Germany) in animals 4–8. To dissect the larger amount of Congo red-positive areas, three samples were additionally collected using 30-gauge needles from animal 7 (800,000 μm^2^) under a stereomicroscope.

The proteomic analysis was conducted as described previously^3,5,6^. The microdissected samples from animals 4–8 were put into 40 μl of lysis buffer (containing 10 mM Tris, 1 mM EDTA, and 0.002% Zwittergent 3–16; Calbiochem, San Diego, CA), boiled for 120 min, and sonicated in a water bath for 60 min. Each sample underwent digestion with trypsin (Mass Spectrometry Grade; FUJIFILM Wako Pure Chemical Corporation, Osaka, Japan) at a concentration of 0.5 mg/ml, maintained at 37 °C for 18 h. Subsequently, the reduction step was carried out using dithiothreitol (FUJIFILM Wako).

LC–MS/MS was performed by high-performance LC interfaced with a mass spectrometer (LTQ Orbitrap XL, Thermo Fisher Scientific, Waltham, MA). The peptides subjected to digestion were separated using an Inertsil ODS-3 column (GL Sciences Inc., Tokyo, Japan) and eluted with a gradient of buffer B (100% acetonitrile and 0.1% formic acid) in buffer A (0.1% formic acid in water).

The LC–MS/MS data were collated with the theoretical fragmentation patterns of peptide sequences in the protein database of leopard gecko (*Eublepharis macularius*, NCBI accession: GCF_028583425) via the Mascot server (Matrix Science Inc., Boston, MA)^48^. As MS/MS ions search conditions, in silico proteolytic enzymes were used “semitrypsin”, and in silico variable modifications were used oxidation of methionine and methylation of lysine. Peptides among the detected ones that exhibited an expectation value of less than 0.05 were deemed statistically significant and are presented in the results.

### Immunohistochemical examination

After the representative proteins were detected by mass spectrometry-based proteomic analysis, immunohistochemical examination was conducted. Immunohistochemical staining was performed when sections were positive for Congo red. An anti-ApoE antibody was produced by Cosmo Bio Co., Ltd (Tokyo, Japan). An antigenic peptide (C + SSVSEKGLQRVQALQQ) derived from the ApoE of the leopard gecko (accession: XP_054855853.1) was designed by the amyloidogenic region (described above). A rabbit was immunized with the peptide, and a high antigen titer was confirmed in the serum. The serum was affinity-purified and used as the primary antibody (dilution 1:4000). In addition, an anti-human cytokeratin 5 rabbit polyclonal antibody (dilution 1:200, GTX113219, GeneTex, Irvine, CA) was included. After antigen retrieval was achieved by a high-pressure steam sterilizer at 121 °C for 10 min using target retrieval solution (Dako North America, Inc., Carpinteria, CA), the sections were incubated with the primary antibody. Subsequently, the sections were covered by Histofine simple stain MAX-PO (MULTI) (NICHIREI BIOSCIENCES INC., Tokyo, Japan) at room temperature for 30 min. The sections were visualized with 3,3′-diaminobenzidine (DAB Tablet; FUJIFILM Wako) and counterstained with Mayer’s hematoxylin. Epithelial cells in internal controls were immunolabeled with anti-cytokeratin 5 antibody, and adequate positivity was observed.

### Supplementary Information

Supplementary Figures.

Supplementary Tables.

## Data Availability

The data analyzed in this study are available from the corresponding author upon request.
