# Supplementary material for: Identification of apolipoprotein E-derived amyloid within cholesterol granulomas of leopard geckos (Eublepharis macularius)
Source: Sci Rep. 2024 Jun 14;14:13746. doi: 10.1038/s41598-024-64643-y (PMC11178906; doi:10.1038/s41598-024-64643-y)
Supplement: Supplementary file 1 — Supplementary Figures. [file 41598_2024_64643_MOESM1_ESM.pdf]

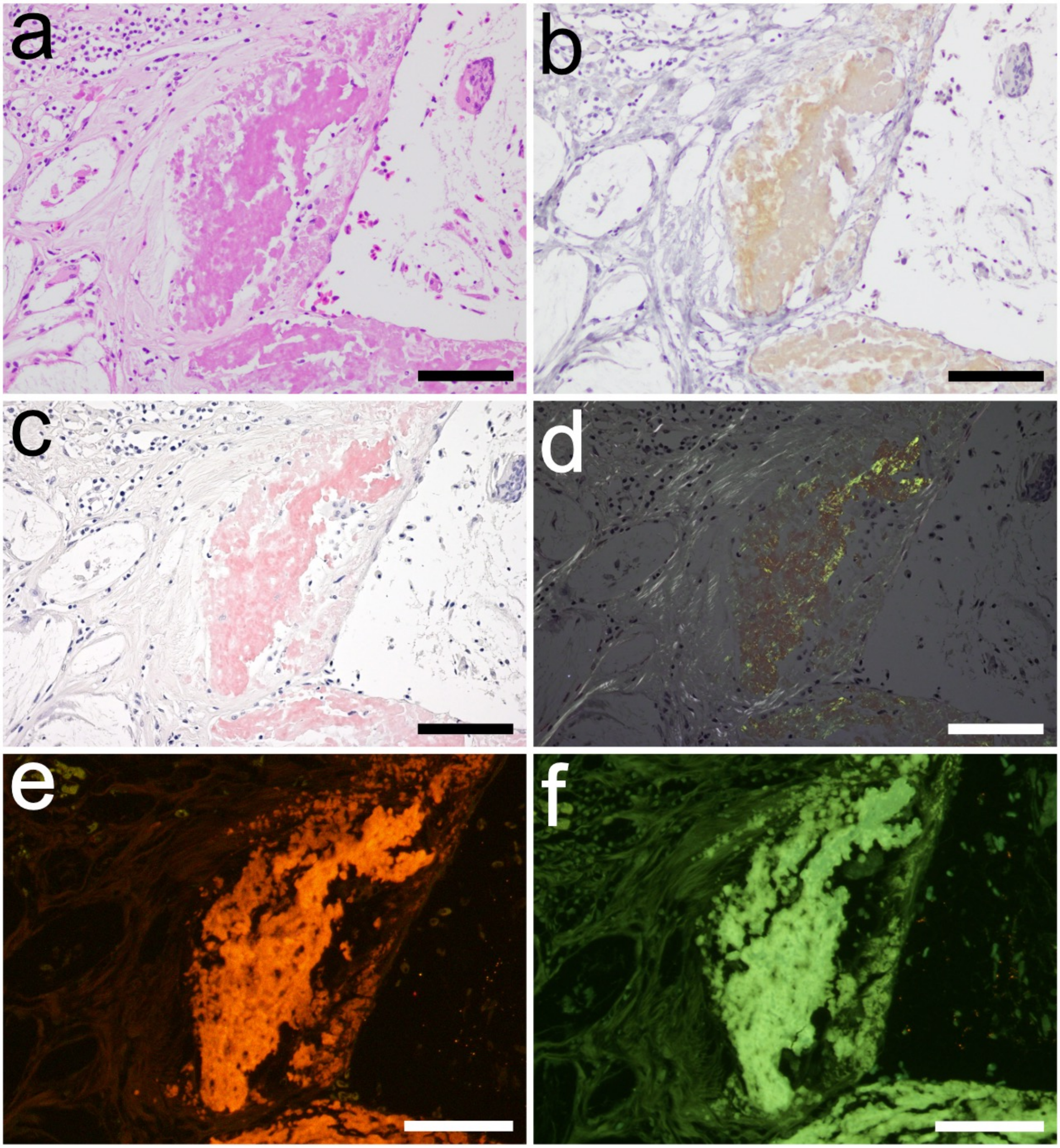

Supplementary Figure S1. Histopathological and immunohistochemical findings of amyloid deposits within cholesterol granulomas of a leopard gecko (Animal 4). Bars = 100 µm. (a) Hematoxylin and eosin staining. (b) Immunohistochemical staining using anti-leopard gecko apolipoprotein E antibodies. (c) Congo red staining. (d) Congo red-stained specimen under polarized light. (e) Congo red-stained specimen under fluorescence microscopy. (f) Thioflavin S staining.

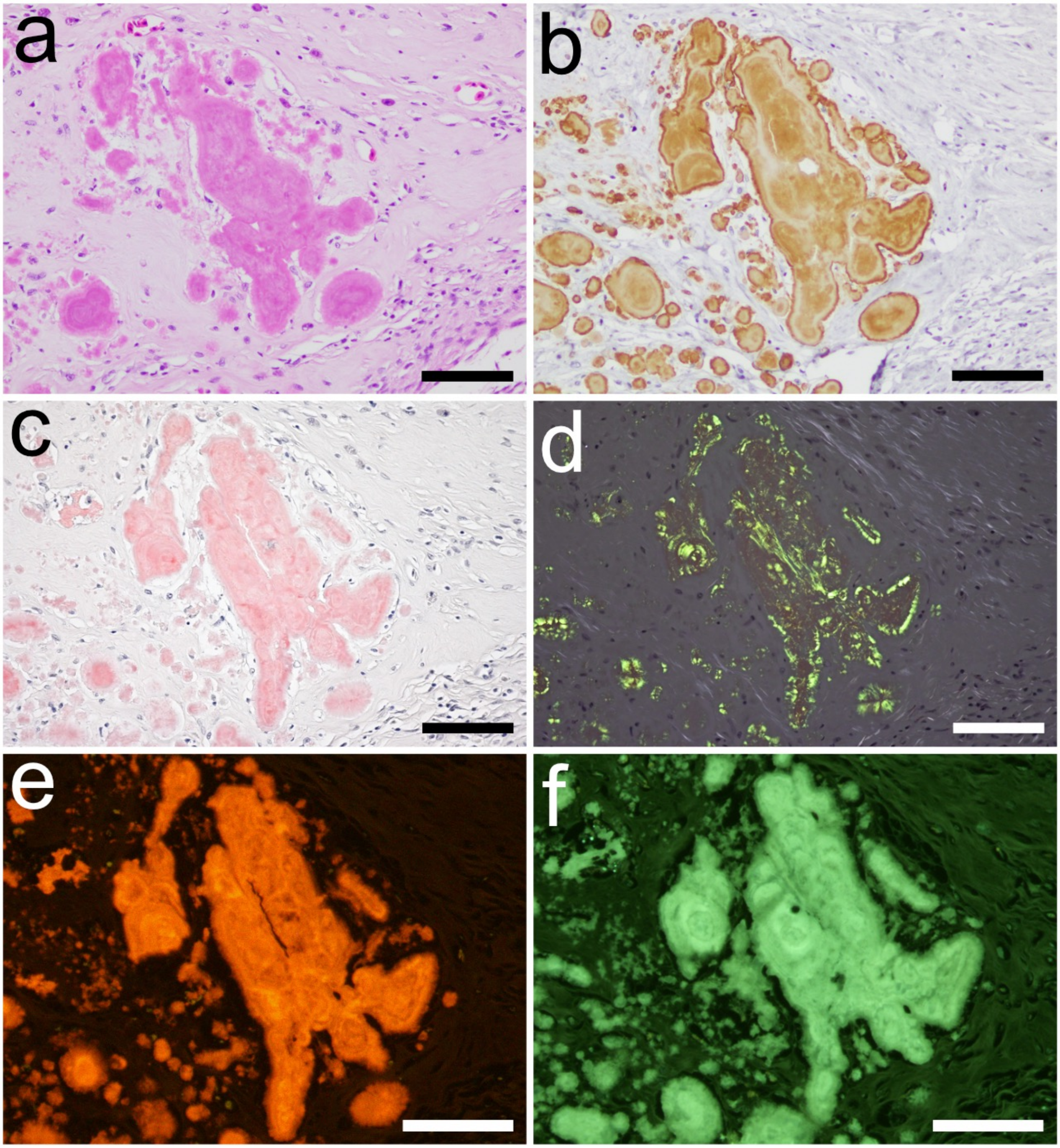

Supplementary Figure S2. Histopathological and immunohistochemical findings of amyloid deposits within cholesterol granulomas of a leopard gecko (Animal 6). Bars = 100 μm. (a) Hematoxylin and eosin staining. (b) Immunohistochemical staining using anti-leopard gecko apolipoprotein E antibodies. (c) Congo red staining. (d) Congo red-stained specimen under polarized light. (e) Congo red-stained specimen under fluorescence microscopy. (f) Thioflavin S staining.

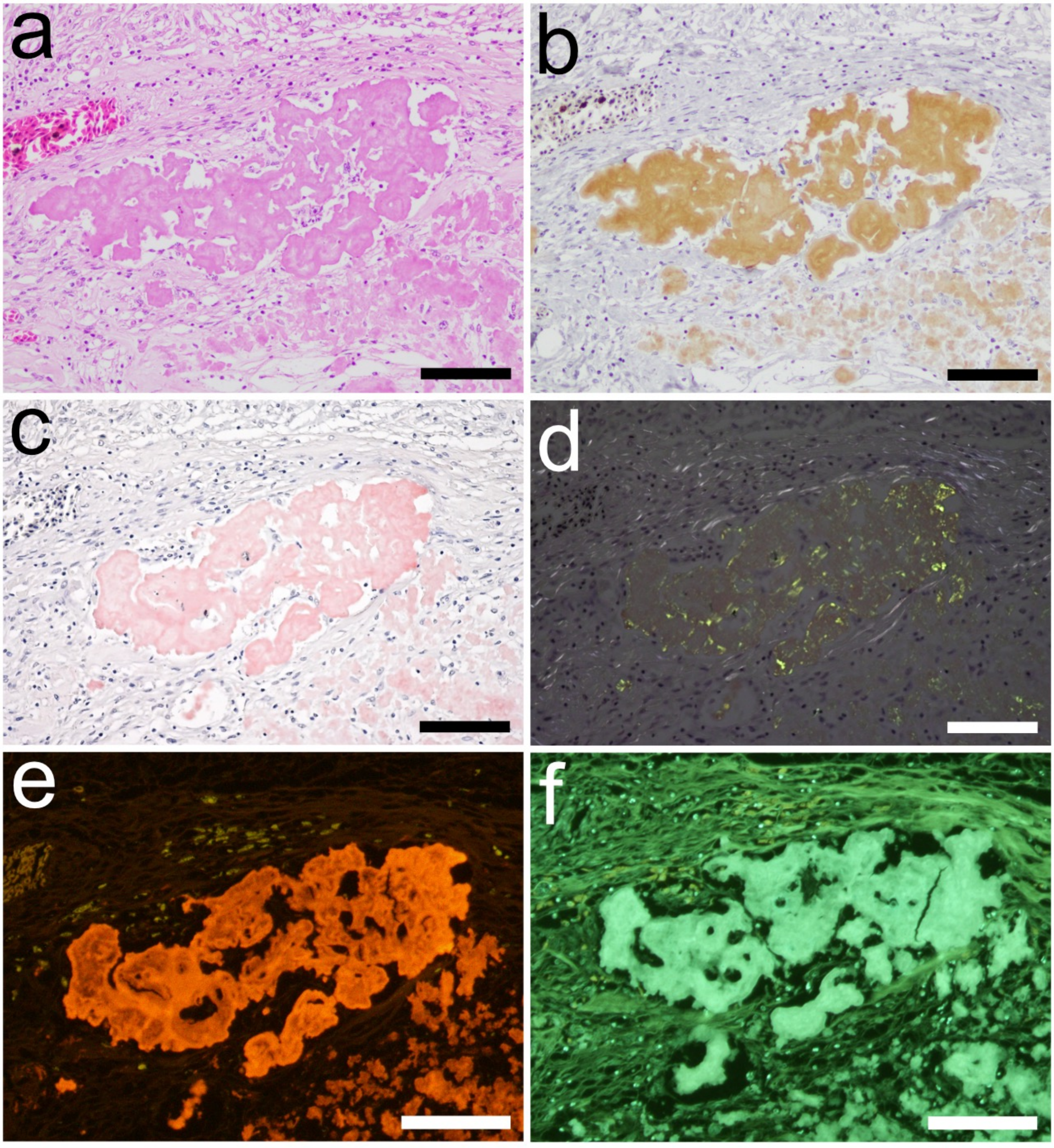

Supplementary Figure S3. Histopathological and immunohistochemical findings of amyloid deposits within cholesterol granulomas of a leopard gecko (Animal 7). Bars = 100 μm. (a) Hematoxylin and eosin staining. (b) Immunohistochemical staining using anti-leopard gecko apolipoprotein E antibodies. (c) Congo red staining. (d) Congo red-stained specimen under polarized light. (e) Congo red-stained specimen under fluorescence microscopy. (f) Thioflavin S staining.

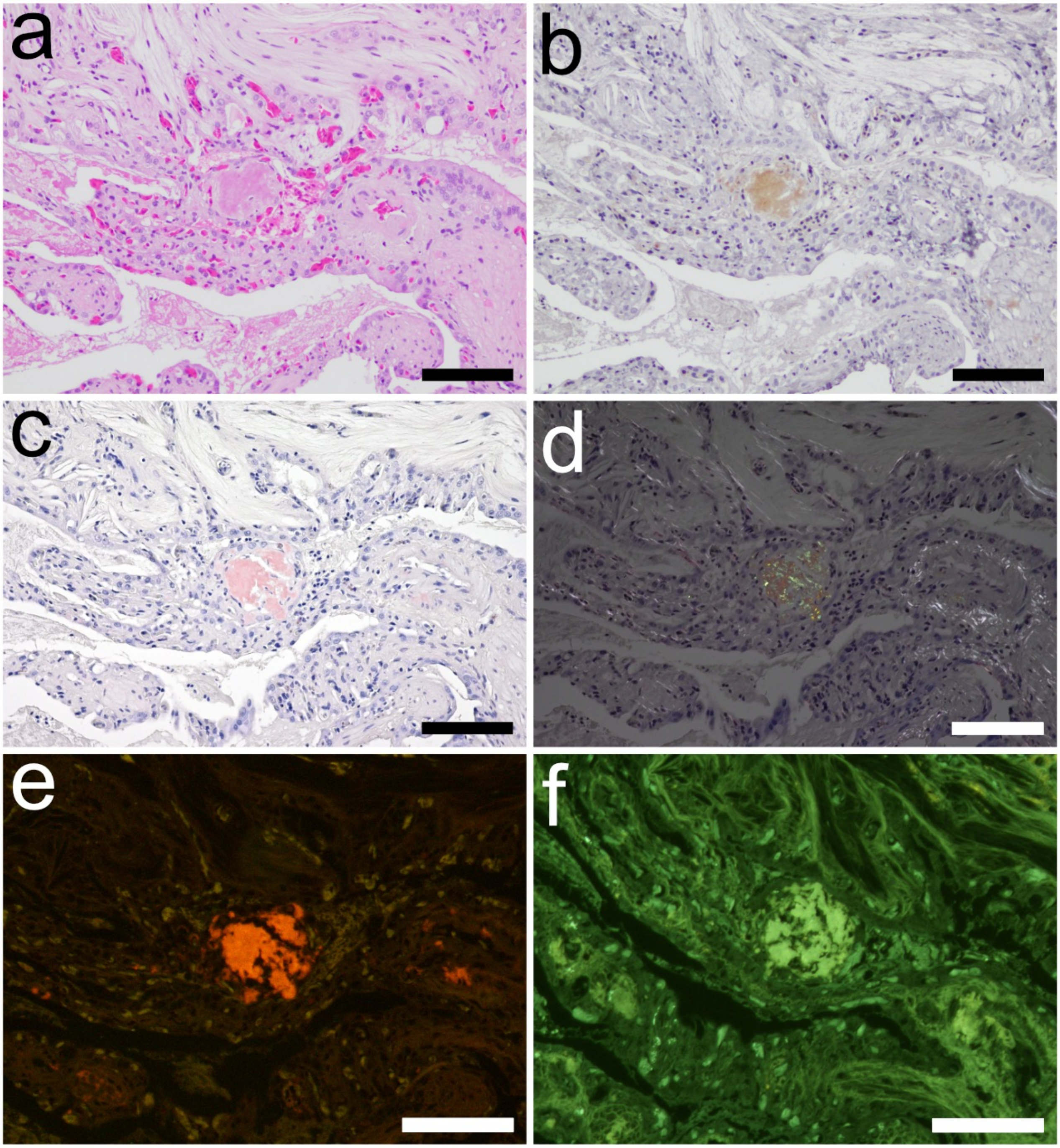

Supplementary Figure S4. Histopathological and immunohistochemical findings of amyloid deposits within cholesterol granulomas of a leopard gecko (Animal 8). Bars = 100 μm. (a) Hematoxylin and eosin staining. (b) Immunohistochemical staining using anti-leopard gecko apolipoprotein E antibodies. (c) Congo red staining. (d) Congo red-stained specimen under polarized light. (e) Congo red-stained specimen under fluorescence microscopy. (f) Thioflavin S staining.
